# A 3D Analysis of Dendritic Solidification and Mosaicity in Ni-Based Single Crystal Superalloys

**DOI:** 10.3390/ma14174904

**Published:** 2021-08-28

**Authors:** Felicitas Scholz, Mustafa Cevik, Philipp Hallensleben, Pascal Thome, Gunther Eggeler, Jan Frenzel

**Affiliations:** Institute for Materials (IFM), Ruhr University Bochum, Universitätsstraße 150, 44801 Bochum, Germany; mustafa.cevik@rub.de (M.C.); philipp.hallensleben@rub.de (P.H.); pascal.thome@rub.de (P.T.); gunther.eggeler@rub.de (G.E.); jan.a.frenzel@rub.de (J.F.)

**Keywords:** single crystal superalloys, seeded Bridgman solidification, competitive dendrite growth, mosaicity, quantitative tomographic characterization

## Abstract

Ni-based single crystal superalloys contain microstructural regions that are separated by low-angle grain boundaries. This gives rise to the phenomenon of mosaicity. In the literature, this type of defect has been associated with the deformation of dendrites during Bridgman solidification. The present study introduces a novel serial sectioning method that allows to rationalize mosaicity on the basis of spatial dendrite growth. Optical wide-field micrographs were taken from a series of cross sections and evaluated using quantitative image analysis. This allowed to explore the growth directions of close to 2500 dendrites in a large specimen volume of approximately 450 mm^3^. The application of tomography in combination with the rotation vector base-line electron back-scatter diffraction method allowed to analyze how small angular differences evolve in the early stages of solidification. It was found that the microstructure consists of dendrites with individual growth directions that deviate up to ≈4° from the average growth direction of all dendrites. Generally, individual dendrite growth directions coincide with crystallographic <001> directions. The quantitative evaluation of the rich data sets obtained with the present method aims at contributing to a better understanding of elementary processes that govern competitive dendrite growth and crystal mosaicity.

## 1. Introduction

Nickel-based single crystal superalloys (SXs) represent key materials for blades in modern gas turbines for power plants and aircraft engines [1,2,3,4,5,6]. They are able to withstand high-temperatures (e.g., >1000 °C) and high mechanical stresses. The creep behavior of SX is of utmost technological importance because it limits life [6,7,8]. It has been demonstrated on various occasions in the literature [6,9,10] that mechanical high-temperature performance strongly depends on microstructures. SX components therefore do not contain grain boundaries as these internal interfaces are prone to the formation of creep pores [6]. SX components are prepared such that the crystallographic <001> direction closely matches the mechanical loading direction. Microstructures of SXs are characterized by large-scale (cast microstructure: dendrites and interdendritic regions) and small-scale (γ/γ′ microstructure) heterogeneities [11,12]. While a relatively good understanding has been established of how the desired type of microstructure can be obtained by processing, the preparation of high-quality superalloy single crystals still remains a difficult task [6,13,14,15,16,17].

SX are prepared by the Bridgman method, where processing parameters need to match a narrow window [17,18]. Different types of defects, e.g., freckles [18,19,20], silvers [21,22] and elongated stray grains [23,24], can form during Bridgman processing of SX. These defects are unwelcome as they affect creep performance. There is one additional defect that has received insufficient attention so far. Technical SXs do not represent single crystals in a strict sense. In fact, they consist of regions that are slightly misoriented with respect to each other and that are separated by low-angle grain boundaries (LAGBs) [25,26,27,28,29]. This defect is referred to as crystal mosaicity [26,27,28,29,30]. The impact of crystal mosaicity on creep behavior has recently been discussed in literature [31,32,33,34]. While it is not clear how far it is relevant for high-temperature mechanical behavior, one has to consider that the key element Rhenium, which is added by low amounts to reduce creep rates (e.g., 3.0 wt.%, [6,35]), strongly segregates to LAGBs in SX [36].

A good overview on crystal mosaicity in SX has been provided in reference [30]. Mosaicity is caused by the deformation of dendrites during solidification [37,38,39,40,41,42,43]. As each dendrite is expected to grow along its crystallographically preferred direction [44], the preparation of a *perfect* single crystal requires that all dendrites grow exactly parallel. In a technical single crystal, however, this is not the case. Dendrite deformation results in small crystal lattice rotations and thus in non-parallel growth, which gives rise to mosaicity. Possible reasons for dendrite deformation have been discussed in [29,41,42,45,46,47,48,49,50,51]. Doherty [29] suggested two categories of dendrite deformation processes, namely, morphological and mechanical bending. Morphological bending occurs when the local chemistry of the liquid phase alters the morphological growth direction of a dendrite without affecting its crystallographic orientation. Corresponding solidification conditions can be established by convection [45]. In contrast, mechanical bending is related to mechanical stresses that cause crystal lattice rotations. It has been documented in the literature [41,42] by combined in situ solidification and radiography experiments using small volume samples that dendrites are mainly deformed by bending. This is in line with findings observed by post-mortem electron back scatter diffraction (EBSD) [37,38,39,42,52]. Recently, direct evidence for dendrite bending and torsion during conventional Bridgman solidification of larger melt volumes has been provided [30]. Several reasons for dendrite deformation have been discussed in the literature, e.g., [29,30,41,43,46,47,48,49,50,51,52,53]. While some studies invoke thermal and shrinkage stresses as main factors [29,46,47,48], others propose strong convective forces [49,50,51], thermomechanical interactions with the casting environment [41,42,43], and precipitation-related phenomena [53]. At present, it is not clear whether one of these possible mechanisms plays a dominant role in the formation of mosaicity in SX.

In the present study, we introduce a novel tomographic approach for the characterization of crystal mosaicity and related dendrite growth phenomena. There is a need to understand the three-dimensional nature of mosaicity, as has been previously highlighted in Reference [30]. Our technique is based on a combination of spark erosion serial sectioning, metallographic preparation, advanced optical microscopy and quantitative image analysis. The tomographic microstructural data allow to reconstruct growth directions of large dendrite numbers across the millimeter and centimeter scale. The obtained data can also be used to characterize the evolution of dendrite neighborhood-relationships, dendrite spacings and aspects related to competitive dendrite growth. The present work differs from other studies where a three-dimensional microstructural characterization was performed, e.g., [54,55,56,57,58,59,60,61]. In fact, most related studies addressed different research objectives, e.g., [51,52,53,54,55,56], or focused on significantly smaller volumes, e.g., [59,60,61].

**Figure 1:**
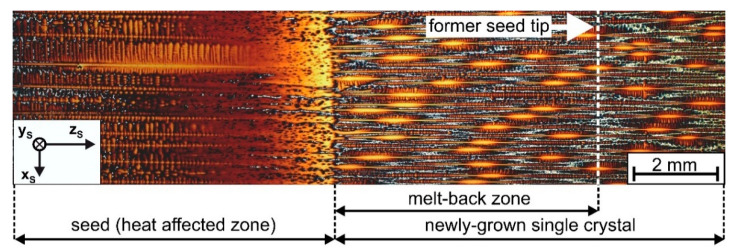
Transition zone between back-melted seed and newly-grown SX (optical micrograph, longitudinal cross section). Modified reprint from Reference [62], with permission from Elsevier. In the present work, a similar sample was investigated by a quantitative three-dimensional tomographic procedure.

A special focus is placed on how mosaicity and dendritic microstructures evolve in the early stages of seeded Bridgman growth. Figure 1 presents a longitudinal section showing the transition zone between a pre-oriented single crystal superalloy seed (left part of image) and a newly grown single crystal (central and right image parts) [62]. During Bridgman processing, the seed melted several millimeters back until the withdrawal process triggered the formation of new dendrites with smaller spacing. Detailed structural, chemical and crystallographic aspects of seeded Bridgman processing were analyzed in our previous work [62]. In the present study, an effort is made to investigate a sample that is comparable to what is shown in Figure 1. The aim is to characterize the spatial evolution of microstructures and mosaicity using our new tomographic procedure.

## 2. Materials and Methods

### 2.1. Crystal Growth/Solidification Experiments

**Figure 2:**
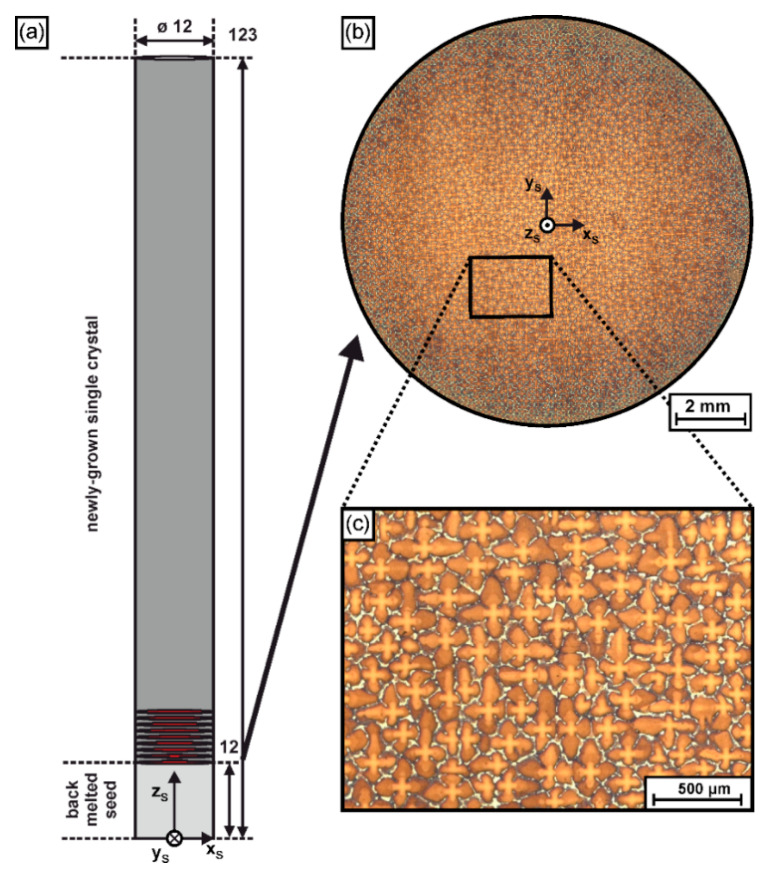
Serial sectioning of the cylindrical SX rods prepared by seeded Bridgman solidification. (**a**) Geometry, coordinate system and positions of cross-sectional planes (red color). (**b**) Example for dendritic microstructures (here: first sample slice of the newly-grown single crystal). (**c**) Microstructure from black rectangular field at higher magnification.

The present work studies the evolution of dendritic microstructures in an as-cast <001> ERBO/1single crystal (a CMSX-4 type alloy [11]) with cylindrical geometry. The SX sample was prepared by seeded Bridgman solidification using a Bridgman furnace of type KZV-A40-400/161G-V operating at a temperature of 1550 °C and imposing a thermal gradient of about 13 K/mm. A withdrawal rate of 180 mm/h was applied. The feedstock material was purchased from Alcoa Howmet (Whitehall, OH, USA). All details on our crystal growth procedure are available in the literature [30,62]. Figure 2 presents geometrical and microstructural details of the as-grown SX ingot. The SX cylinder is characterized by a diameter of 12 mm and ≈120 mm length, Figure 2a. The lower part of the sample (light grey region in Figure 2a) represents the seed. During Bridgman processing, the seed was melted back from an initial length of 16 mm to ≈11 mm, from where the newly-grown crystal formed by epitaxial growth during withdrawal. Figure 2a specifies the position where a series of cross section samples, indicated as red slices/disks, was extracted for tomographic analysis. The corresponding samples allowed to characterize the evolution of microstructures from an early solidification stage where new dendrites formed from the back-melted seed, Figure 1. Figure 2b,c exemplarily show typical dendritic microstructures that can be observed in cross sections of the SX cylinder. The SX bar contains a large number of individual dendrites, and the average dendrite spacing is close to 200 µm. Figure 2a,b provides a sample coordinate system, where the z_S_ axis matches the longitudinal axis of the SX cylinder. This x_S_-y_S_-z_S_ coordinate system allows to specify positions of specific tomographic samples, as well as individual dendrite positions.

### 2.2. Tomographic Characterization, Metallographic Preparation and Crystallographic Analysis

*Tomographic procedure.* The basic workflow for tomographic characterization involves four steps. (1) Disk-shaped samples (red cross sections in Figure 2a) were extracted from defined positions of the SX cylinder and prepared for metallographic analysis. (2) An advanced optical microscopy system was used to retrieve detailed wide-field micrographs, which cover complete cross sections of the SX cylinder. (3) All micrographs originating from the various sample slices were registered with respect to the coordinate system, Figure 2a, using image processing software. (4) The tomographic microstructural data were evaluated and interpreted in terms of dendrite positions, dendrite growth directions and other microstructural features.

*Metallographic preparation:* SX slices were successively extracted from the cylindrical ingot by electro discharge machining (EDM), applying incremental z_S_ steps of 1mm. For metallographic preparation, an automated grinding and polishing system of type Tegrapol 15 (Struers GmbH, Willich, Germany) was applied. Polished samples were etched using a solution of 100 mL H_2_O, 100 mL HCl, 100 mL HNO_3_ and 3g MoO_3_. This preparation procedure establishes good chemical contrast, which allows to appreciate the dendritic microstructure. Further details on the metallographic procedure are given elsewhere [30,62]. In the present work, an effort was made to precisely evaluate geometrical changes of each specimen. As EDM cutting and metallographic preparation (only one side was polished) are associated with materials loss, precise sample thickness data were required to relate the tomographic micrographs to exact z_S_ positions in the SX bar. The final thickness values of all sample slices were carefully evaluated throughout the complete metallographic preparation routine by using a dial indicator gauge of type ID-H0530 (Mitutoyo Corp., Neuss, Germany). The total materials loss varied between 0.5 and 0.6 mm on the polished side of each sample. A total number of 9 slices was studied in the present work.

*Automated wide-field image acquisition:* An optical microscope of type Axio (Carl Zeiss GmbH, Oberkochen, Germany) equipped with high-quality lenses, a high-resolution CCD camera of type Leica DFC320 and a stepper-motor-driven sample stage of type Tango Desktop (Märzhäuser, Wetzlar, Germany) was used to automatically generate large mosaics of micrographs that completely cover the sample cross sections, Figure 2b. For each mosaic, a total number of at least 50 overlapping image tiles was acquired and automatically stitched together using the software package Imagic ims (company Imagic Bildverarbeitung AG, Opfikon, Switzerland) [63]. An optical coordinate-reading microscope of type WMZ (Hitech Meßtechnik GmbH, Magdeburg, Germany) was used to verify whether the applied procedure performed well in image stitching. A direct comparison between cross section dimensions obtained from the final stitched image and reference values, which were directly measured using the coordinate-reading microscope, deviated by less than 0.21% (close to 25 µm). This is acceptable for the scope of the present work. No effort was made to correct optical distortions of the microcopy system as they were sufficiently low.

**Figure 3:**
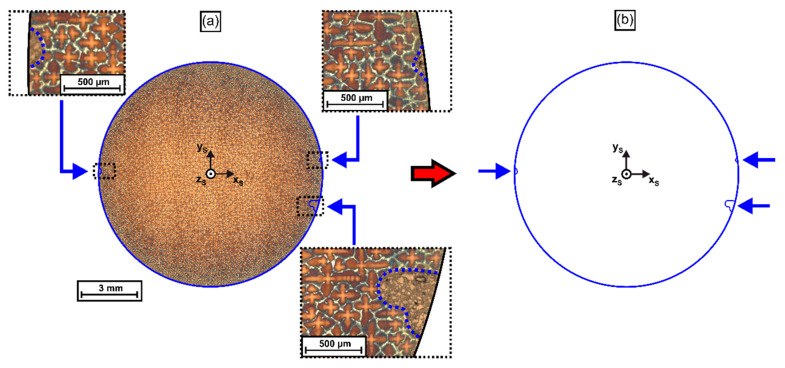
Generation of a digital reference mask for the registration of tomographic micrographs taken at different height positions (z_S_) of the SX rod. (**a**) Sample cross section with three EDM marker notches. (**b**) Corresponding digital reference mask. The blue arrows indicate the positions of the three EDM marker notches.

**Figure 4:**
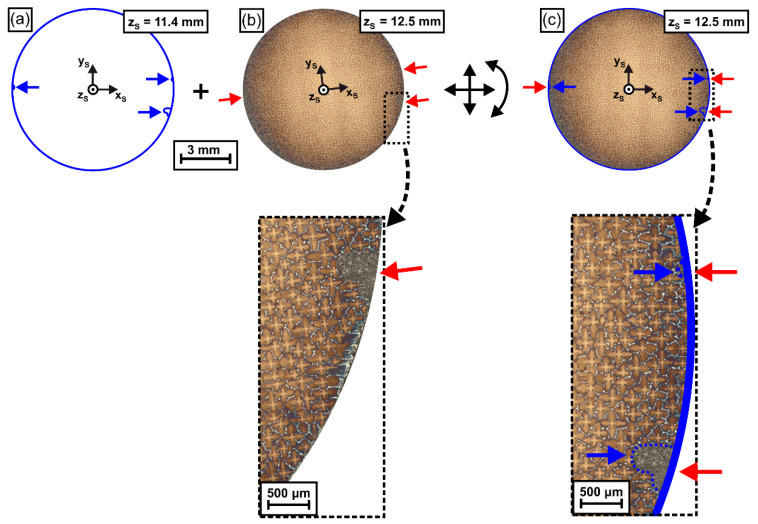
Alignment/registration of sample slices from different height positions using the reference mask. (**a**) Reference mask from first sample slice, see Figure 3. (**b**) Raw image from a slice extracted from a higher z_S_ position. (**c**) Same image as in (**b**) after alignment using the reference mask (**a**). The high magnification micrographs document that a good match between image and reference mask was obtained. Blue arrows: EDM notches in reference mask. Red arrows: EDM notches in target image. For details see text.

*Image registration:* A mask-based image processing technique was applied to precisely align/register all tomographic images with respect to the x_S_-y_S_-z_S_ coordinate system, Figure 2a, and to avoid image rotations. The procedure invokes microscopic reference markers. Prior to slicing, these markers were applied by establishing longitudinal groves in the shell surface of the cylindrical SX bar through EDM processing. The depth of these marks ranged between 0.1 and 0.5 mm, such that they could be detected in each cross-section micrograph. In our image processing routine, the first micrograph (height z_S_ = 11.4 mm) served as a reference for image registration. This image slice was used to generate a contour mask by manual drawing operations using the graphic and image software package CorelDraw [64]. All other micrographs were registered by using this mask, as illustrated in Figure 3 and Figure 4. Figure 3a presents an overview image of the reference image slice where the three marker groves are shown at higher magnification. The markers are indicated by blue dashed lines. However, it is a little difficult to spot their contours as they are filled with metallographic mounting material. We note that the first image slice/reference mask was oriented such that the x_S_ and y_S_ axes are aligned parallel to the secondary dendrite arms. Figure 3b shows the isolated mask (blue arrows: marker groves in Figure 3a).

In a next step, the reference mask was used to register/align all micrographs (obtained from different ingot height positions) with respect to the x_S_-y_S_-z_S_ coordinate system, Figure 4. Figure 4 exemplarily illustrates how the mask, Figure 4a, is applied to adapt the position and rotation angle of a micrograph obtained from a height of z_S_ = 12.5 mm, Figure 4b. The final registered image is shown in Figure 4c. Image registration was performed such that the marker notches of the contour mask (blue arrows) and related notches in each tomographic image (red arrows) matched. The inset in Figure 4b shows a situation from an early stage of image registration, where mask and image notches do not yet coincide as only one image notch (red arrow) is visible. In contrast, Figure 4c documents a good alignment where mask and image notches perfectly match. This procedure was applied to all tomographic images (see Figure 2a) obtained in the present work.

**Figure 5:**
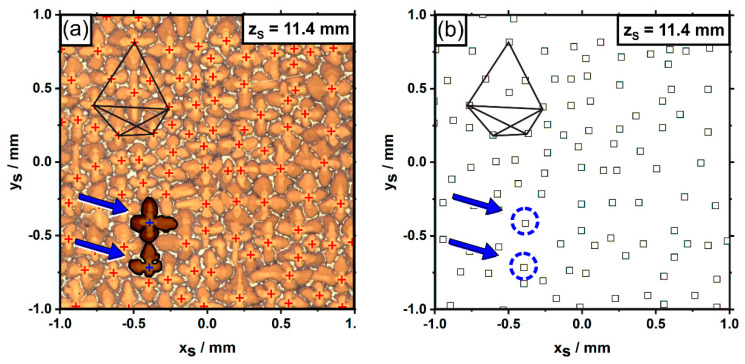
Example for the extraction of primary dendrite arm positions (z_S_ = 11.4 mm). (**a**) Optical micrograph. Red crosses: positions where coordinates of primary dendrite arm centroids were retrieved. (**b**) Isolated representation of dendrite positions. A polygonal dendrite constellation serves as reference. The blue arrows highlight two misaligned dendrites.

*Data analysis and visualization:* Two different approaches for data analysis and visualization were applied in the present study. The first approach aims for a reconstruction of dendrite growth directions. Central positions of primary dendrite arms were determined from registered images by manual coordinate extraction using the software package ImageJ [65], followed by a subsequent transformation into x_S_-y_S_-z_S_ sample coordinates, Figure 2a. An example for this procedure is presented in Figure 5. Figure 5a shows a small part of a micrograph from the first sample slice, corresponding to a z_S_ position of 11.4 mm. The small red crosses indicate centroids of primary dendrite arms growing opposite to the viewing direction. A dendrite constellation with polygonal shape is illustrated in the upper left for reference. The blue arrows point to two specific dendrites that will be considered in detail at a later stage. Figure 5b presents a different representation of the sample region in Figure 5a. Figure 5b exclusively contains x_S_-y_S_ dendrite positions that were retrieved following the procedure described above. The type of data extraction presented in Figure 5 was applied to the first five slices that were cut out from the SX bar at different heights positions (Figure 2a). The resulting data sets contained a total number of 13,380 dendrite positions. These coordinates were used to manually reconstruct all individual dendrite growth paths within the first 4 mm of the newly-grown SX, Figure 2a. All registered image data and dendrite positions are available to the public in the open-access data repository zenodo (link in reference [66]). We hope that these data can be useful for other research studies on solidification.

**Figure 6:**
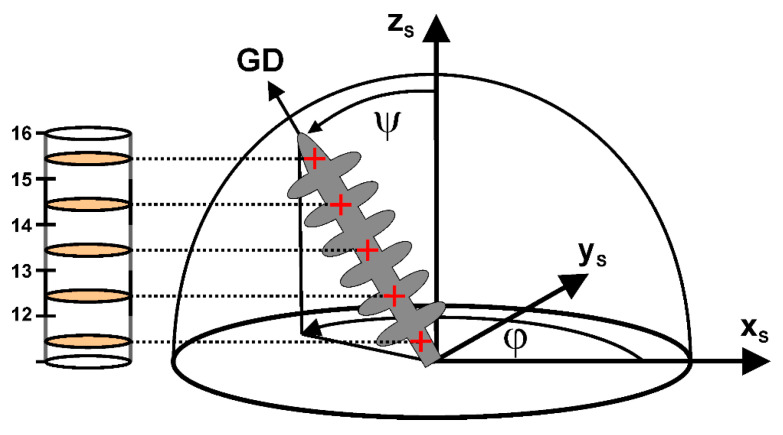
Determination of growth directions for individual dendrites from tomographic image slices. The growth direction (GD) is expressed through polar ψ and azimuth angles φ. For details, see text.

Figure 6 schematically shows how growth directions of all individual dendrites were evaluated by applying a linear fit procedure. Figure 6 illustrates a scenario where a single dendrite grows from the bottom towards the upper image part. The growth direction deviates from the z_S_ axis (longitudinal axis of the cylindrical SX), which represents the ideal/target growth direction. Figure 6 shows a linear fit of the central dendrite positions (marked in red) across several image slices. The growth direction finally can be expressed by a combination of a polar angle ψ and an azimuth angle φ, both indicated in Figure 6. The two angles ψ and φ represent key parameters that were used to generate color-coded orientation distribution maps.

**Figure 7:**
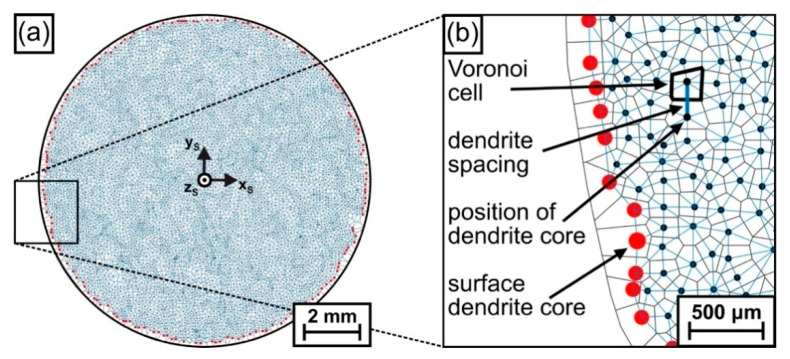
Characterization of microstructures using Voronoi tessellation [67,68]. (**a**) Overview image. (**b**) All dendrite positions in the spherical disk are represented by dark blue points. The red points mark the position of dendrites on the outer rim of the sample. For details, see text.

*Voronoi tessellation:* As a second technique for data analysis, Voronoi tessellation [67,68] was applied to describe the evolution of dendritic microstructures in terms of the numbers of nearest dendrite neighbors and primary dendrite arm spacings. The Voronoi algorithms implemented in the software toolbox MTEX [69,70] in MATLAB [71] were used to generate two-dimensional Voronoi cells based on the dendrite coordinates retrieved from each specimen slice. Figure 7 exemplarily shows how this technique was used to evaluate the microstructure of the first image slice (z_S_ = 11.4 mm, see Figure 2b for reference). Figure 7a shows a graphical representation of all dendrite positions after Voronoi processing. A small part of this image is presented in Figure 7b at a higher magnification. In Figure 7b, the dark blue and red dots indicate central positions of primary dendrite arms, whereas dark blue dots are related to “regular” dendrites that are surrounded by other dendrites. In contrast, red dots indicate dendrites that are located at the shell surface of the ingot. The black lines in Figure 7b indicate Voronoi cell boundaries, and the blue lines, which were determined by Delaunay triangulation [68,72], represent the distances between nearest-neighbor dendrites (corresponding to local primary dendrite arm spacings). In the present work, the arrangement of dendrites was characterized by four parameters, namely, by individual, maximum, minimum and average spacings of each single Voronoi cell.

*Crystal orientations:* A new high-angle resolution orientation image mapping (OIM) technique—rotation vector base-line electron back scatter diffraction (RVB-EBSD) [52]—was applied to determine crystallographic orientations. RVB-EBSD analysis was performed using a scanning electron microscope (SEM) of type FEI Quanta FEG 650 equipped with an EBSD detector of type EDAX Ametek Hikari. Data were collected at 30 kV and a step size of 1 μm. Kikuchi patterns were recorded with 1 × 1 binning at a resolution of 512 × 512 pixels. The diffraction patterns were analyzed with the RVB-EBSD method, which is explained elsewhere in detail [52]. The method was based on a cross-correlation procedure, which was performed on 48 regions of interest on each Kikuchi pattern (~630,000 patterns in total). The angular resolution was approximately 0.03°. We provide different methods of visualization, which allow to appreciate the different components of the orientation data. For details, see reference [52].

## 3. Results

### 3.1. Dendrite Growth Directions

**Figure 8:**
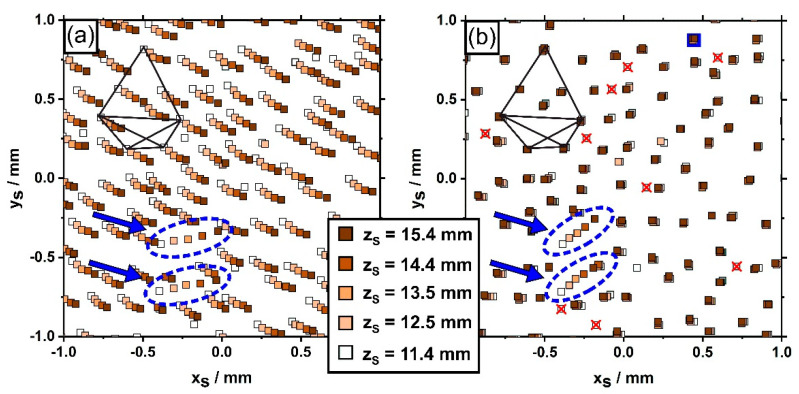
Color-coded microstructural data that show the evolution of dendrite core positions during 4 mm growth (from z_S_ = 11.4 mm to z_S_ = 15.4 mm) (**a**) Raw data. (**b**) Same microstructural data as presented in (**a**) after alignment. The black polygonal reference group represents the same dendrites as in Figure 5a. The blue arrows highlight two dendrites which do not follow the average growth trend. Small red crosses in (**b**) indicate dendrites that were no longer detected after z_S_ = 11.4 mm. The blue square in the upper right is related to one dendrite which newly formed during the first 1.1 mm growth.

Figure 8 exemplarily shows how the microstructure of a small part of the cylindrical SX specimen evolves between the first (z_S_ = 11.4 mm) and the fifth slice (z_S_ = 15.4 mm). All data points in Figure 8a represent unprocessed dendrite positions plotted in a x_S_-y_S_ coordinate grid. The different colors indicate height positions (z_S_ coordinates) that are specified by the color code in the legend. The region in Figure 8 matches the sample part considered in Figure 5 (see polygonal dendrite constellation for reference). The data in Figure 8a allow to appreciate that individual data points, which are aligned in a series, are related to one and the same dendrite that grew from the lower towards the upper z_S_ position during solidification. One can easily see that most of the dendrites in Figure 8a do not exactly grow parallel to the longitudinal axis of the SX bar. A perfect parallel growth would have resulted in identical coordinates for each dendrite in all 5 image slices. However, this is not the case. In fact, all x_S_-y_S_ coordinates are shifted, which indicates that the SX sample has a slight overall misorientation. This detail is less important for the objective of the present study. Figure 8a shows a striking phenomenon. Two isolated dendrites marked by blue arrows do not follow the average growth trend. Their projected growth directions point to the right/upper right of the image. This behavior becomes even more prominent when the aforementioned general misorientation of the whole dendrite field is compensated through a mathematical correction. Figure 8b presents the corresponding adjusted dendrite growth data. The corrective step succeeded in virtually eliminating the average misorientation since the data points from the majority of all dendrites coincide as one moves from the first to the fifth z_S_ position, i.e., almost the entire dendrite field now virtually grows parallel to the z_S_ axis/perpendicular to the viewing plane. The data in Figure 8b allow to identify one important phenomenon that characterizes the microstructural evolution in the early stages of seeded Bridgman processing. Several dendrites are present, highlighted by red crosses, that could be exclusively detected in the first two image slices (z_S_ = 11.4 and 12.5 mm). These dendrites did not manage to successfully compete with neighboring dendrites during on-going solidification and thus to reach higher lengths. Only one single new dendrite, which was not present in the first image slice, formed by a branching process [37,38] on the second slice (zs = 12.5 mm). This dendrite is indicated by a blue square in the upper part of Figure 8b.

**Figure 9:**
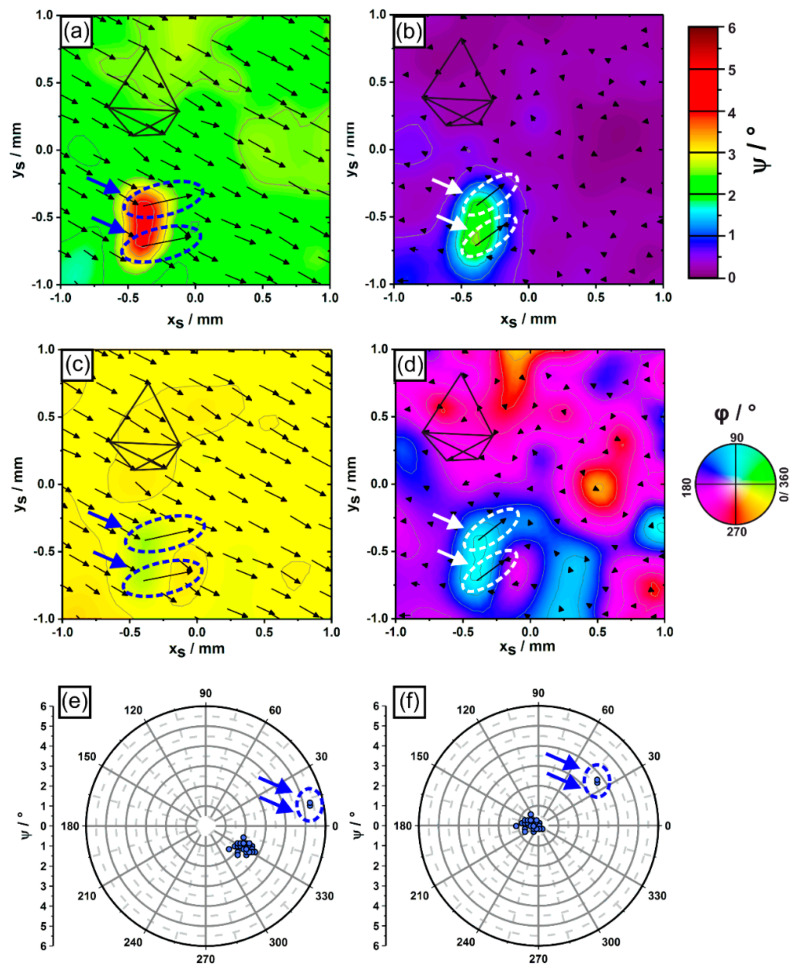
Dendrite growth directions represented by (**a**–**d**) color-coded contour maps showing local distributions of (**a**,**b**) polar and (**c**,**d**) azimuth angles, and by (**e**,**f**) pole figures. Left sub-figures (**a**,**c**,**e**): as-measured dendrite growth directions. Right image part (**b**,**d**,**f**): growth directions after compensation of the overall misalignment. The two arrows highlight two outliers. For details, see text.

The results on dendrite growth presented in Figure 8 were evaluated in terms of polar and azimuth angles. Figure 9 presents corresponding color-coded microstructural data of the same region previously shown in Figure 5 and Figure 8. In Figure 9, the local distributions of dendrite polar angles ψ (Figure 9a,b) and azimuth angles φ (Figure 9c,d) are visualized using color codes specified on the right. In addition, thin black arrows indicate projected x_S_-y_S_ shifts of all dendrites in this region during 4 mm solidification growth. Short/long arrows are related to small/large projected x_S_-y_S_ shifts. We note that Figure 9 exclusively relies on dendrites which managed to persist from the first to the fifth sample slice. All other dendrites were not taken into account. Similar to what was previously shown in Figure 8, the color-coded data in Figure 9a indicates an overall misorientation of about 2° in terms of polar angles. As a striking result, the growth behavior of the two misaligned dendrites differs as they show polar angles close to 5°. Figure 9b shows how the local distribution of polar angles changes when the average misorientation of the whole sample is compensated as described previously. In Figure 9b, one can clearly see that the virtual growth directions of the two misaligned dendrites deviate by more than 2° from the average growth direction of the surrounding dendrite matrix. Figure 9c,d present dendrite orientations in terms of azimuth angles of the same sample region considered above. Most parts of the regions in Figure 9c have a yellow color tone, which indicates that most dendrites have azimuth angles close to 330°, i.e., their projected growth directions point towards the lower right of the image. Again, the two slightly misaligned dendrites show a different behavior as they are represented by green color tones, which indicates that their azimuth angles are close to 15°. The situation also changes for the azimuth plots when the average growth direction is again compensated for. In this case, strong color shifts can be detected throughout the whole sample region. We note that the data in Figure 9d are presented for the sake of completeness to provide full information on growth directions. However, one has to keep in mind that due to a mathematical singularity (see definition of azimuth angles in Figure 6), the azimuth angle is not defined when dendrite growth directions are exactly parallel to the central specimen axis. Small deviations from the idealized growth direction, which can also be related to experimental scatter, therefore cause high fluctuations of azimuth angles. Only dendrites with larger polar angles (e.g., the two misaligned dendrites) yield meaningful azimuth angle values.

**Figure 10:**
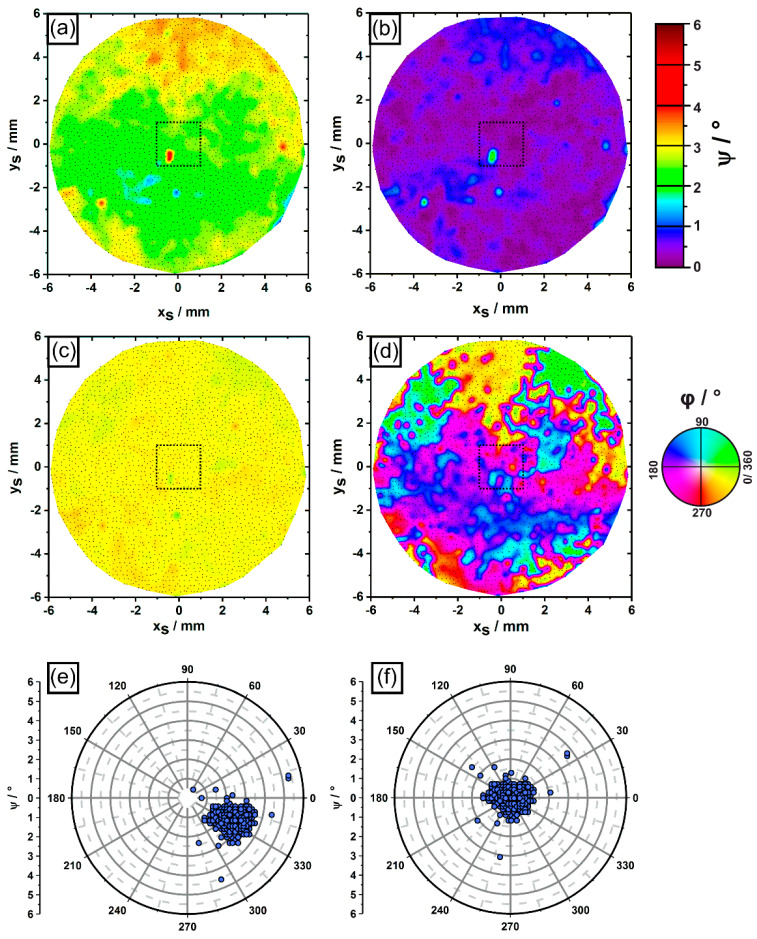
Dendrite orientations/growth directions from whole cross section. (**a**–**d**) Color-coded maps showing (**a**,**b**) polar angle and (**c**,**d**) azimuth angle distributions. The black rectangle indicates the smaller sample region considered in Figure 5, Figure 8 and Figure 9. (**e**,**f**) Pole figure plots of dendrite growth directions. Sub-figures in left column (**a**,**c**,**e**): Raw orientation data. Subfigures in right column (**b**,**d**,**f**): dendrite orientations after compensation of average dendrite misorientation.

The corresponding polar and azimuth angles can be used to generate pole figure plots, Figure 9e,f. Corresponding pole figures revealing the as-measured growth directions are presented in Figure 9e, whereas virtual growth directions obtained after applying the compensation described above are shown in Figure 10f. The larger cluster of data points in both pole figure plots is related to the dendrite matrix. In contrast, the two isolated data points highlighted by arrows represent the two misaligned dendrites.

Figure 10 provides the same type of information previously presented in Figure 9. However, the data comprise the *complete* cross section of the cylindrical ingot. Figure 10 relies on tomographic information retrieved from a sample volume of 450 mm^3^ containing 2479 dendrites, which grew from the first to the fifth image slice. In fact, 2479 out of 2892 dendrites (85.72 %) contained in the first image slice managed to reach a z_S_ height of 15.4 mm. These dendrites are indicated by small black dots in Figure 10a–d. The smaller sample region previously considered in Figure 5, Figure 8 and Figure 9 is marked for reference by a dashed square in the center of the cross section. The left row of sub-figures in Figure 10 (Figure 10a,c,e) relies on as-measured growth directions, whereas the right row (Figure 10b,d,f) is again related to the orientation data after a compensation of the average dendrite misorientation. The following key findings were identified: individual growth directions of dendrites can differ by up to 4° from the average growth direction, Figure 10e,f. Dendrites with stronger misorientations are rare. In fact, 6 out of 2479 dendrites (0.24%) deviate by more than 2° from the average growth direction. It is possible to identify larger misoriented regions with sizes of several mm^2^ (e.g., upper part of Figure 10a), as well as small regions that only consist of 1–3 individual dendrites. The latter ones appear as spot-like features in the color-coded plots in Figure 10a,b. The azimuth angle plots in Figure 10c,d provide similar information to what was previously presented for the smaller sample section in Figure 9c,d. Again, the adjusted data in Figure 10c need to be interpreted with care, as mentioned above. The pole figure plots in Figure 10e,f indicate that the variation of dendrite growth directions of the whole sample field is larger than the smaller sample region considered in Figure 9e,f.

### 3.2. Crystal Orientations

**Figure 11:**
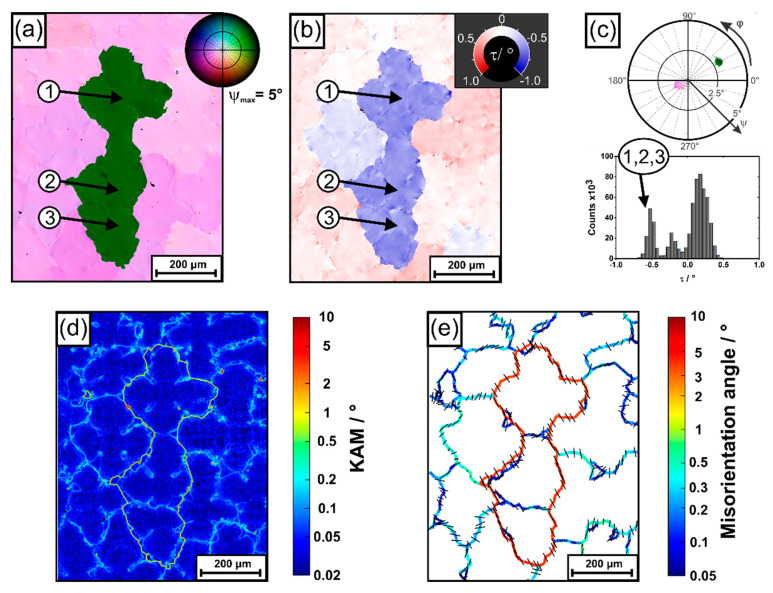
Crystallographic orientations obtained by RVB-EBD from the region considered in Figure 5, Figure 8 and Figure 9. (**a**) Pole figure color coding of <001> directions. (**b**) Rotations around <001> axes. (**c**) Orientation distributions. (**d**) Kernel average misorientation data. (**e**) Misorientations between dendrites characterized through the axis/angle convention. The black bars correspond to projected rotation axes. For details, see text.

So far, the data presented in Figure 8, Figure 9 and Figure 10 reflect *morphological* growth directions of dendrites. In a next step, RVB-EBSD [52] was applied to evaluate *crystallographic* orientations, Figure 11. RVB-EBSD represents a cross-correlation based EBSD method that provides a significantly improved angular resolution, better than 0.03° [52]. It includes the application of a new color-coding that was specifically designed for the orientation imaging of directionally solidified dendritic microstructures. Splitting the orientation information into two components allows to overcome the drawbacks of standard IPF color coding, which fails to completely describe crystallographic growth directions of dendrites in materials with high crystal symmetry. This has been discussed in Reference [52] in detail. As a first type of visualization, we use a pole figure color-coding (as opposed to a conventional inverse pole figure (IPF) color coding) that provides information on the same geometrical basis as described for morphological dendrite growth directions. Since each Kikuchi pattern for EBSD analysis contains the complete 3D orientation information, a second type of visualization is applied to explore rotations around the <001> direction. This rotation can be interpreted as a result from dendrite torsion. A high-angular resolution OIM obtained from a RVB-EBSD scan is presented in Figure 11a. The region covered in Figure 11a contains the same misaligned dendrites previously shown in Figure 8 and Figure 9; however, the size of the corresponding region is slightly smaller. The visualization of the crystallographic orientation data in Figure 10a relies on the previously mentioned pole figure color coding of the <001> direction, as specified in the upper right. Different color tones indicate rotations of the <001> direction away from the viewing direction (central bright area in pole figure), whereas the angular amounts of these shifts are represented by different lightness and saturation values (small/large shifts: bright/dark lightness intensities). One important observation can be made in Figure 10a: the crystal orientations of the misaligned dendrites significantly differ from those of the surrounding dendrite field. This is in line with what was previously shown for data obtained by tomographic growth direction analysis, Figure 8, Figure 9 and Figure 10. However, there is one striking difference. While the tomographic analysis, Figure 8, Figure 9 and Figure 10, allowed to identify two misaligned dendrites, the RVB-EBSD results suggest that actually *three* misaligned dendrites are present in the first image slice. These three dendrites are marked with arrows in Figure 10a. It is a little tedious to identify these three dendrites as dendritic/interdendritic features only provide weak contrast in the OIM data; however, a direct comparison with the optical micrograph, Figure 5a, clearly allows to conclude that the first image slice in fact contains three misaligned dendrites with identical orientation, Figure 11a. The third dendrite could not be detected by tomography as it was only present in the image slice from the first z_S_ position, see corresponding red cross in Figure 8b.

While Figure 11a provides crystallographic data related to *growth directions*, Figure 11b considers *the torsion component* of the orientation, i.e., rotations around the <001> direction of each OIM data point. According to the color-code provided in the upper right of Figure 11b, positive rotations/τ angles (“to the left”) are indicated by red color-tones, whereas negative rotations (“to the right”) are associated with blue colors. In general, the observed torsion component yields angles lower than 1°, which is significantly smaller than what was previously observed for crystallographic growth directions, Figure 11a. Figure 11b allows to conclude that the region has a heterogeneous character. As a striking observation, the τ angles of the three misaligned dendrites significantly deviate from those of the surrounding dendrite field. Figure 11c summarizes crystallographic orientation data using a pole figure plot as in Figure 11a, as well as a histogram of torsion angles as shown in Figure 11b. The most important observation is that the pole figure plot in Figure 11c almost exactly matches the morphological dendrite growth data presented in Figure 9c. This striking similarity allows to conclude that morphological growth directions of dendrites and crystallographic <001> directions are identical. Furthermore, torsion rotation angles are relatively low. They are distributed within ±0.5° around the average value. The histogram in Figure 11c shows three maxima. The first maximum (−0.5°) is related to the three aforementioned dendrites (“1,2,3”).

The obtained RVB-EBSD data allow for a detailed analysis of crystallographic *misorientations* between dendritic regions. Figure 11c presents a color-coded plot of kernel average misorientation (KAM) values. The corresponding data were retrieved by RVB-EBSD analysis considering second order neighborhood [73]. KAM data in Figure 11d suggest that sharp interfaces exist between misoriented dendrites, which thus can be considered as LAGBs (we follow the common convention where grain boundaries with misorientation angles lower than 15° are classified as LAGBs). The color-coded KAM data also allow to identify additional dendritic and interdendritic microstructural features. These details have been discussed in our previous work [52], where an attempt was made to invoke geometrically necessary dislocations for interpretation. The nature of LAGBs between dendrites can be interpreted using the misorientation axes and angles convention [73]. A plot where projected misorientation axes are indicated as black lines and where local misorientation angles are presented using a rainbow-type color code is shown in Figure 11e. The data allow to conclude that actually all dendrites are characterized by small crystallographic misorientations and that they all are separated by LAGBs. In most cases, the misorientation angles are low, e.g., smaller than 0.5°. However, in the case of the three more severely misaligned dendrites, these misorientation angles exceed 2.5°. The projected misorientation axes (small black bars) in Figure 11e indicate that rotation axes appear to be more or less random for most LAGBs. However, most importantly, the rotation axes, which characterize the misorientation between the three more severely misaligned dendrites and their neighbor dendrites, are oriented such that the <001> directions of the three dendrites point to the upper right in the pole figure in Figure 11c.

### 3.3. Evolution of Dendrite Patterns

**Figure 12:**
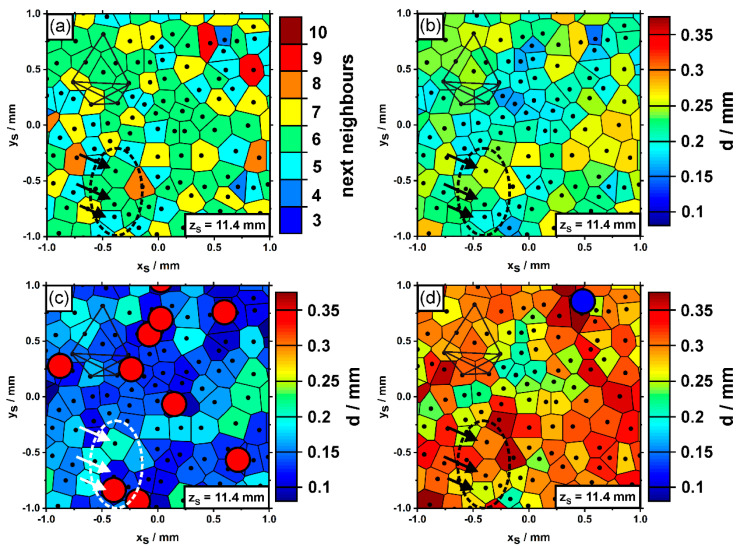
Voronoi tessellation plots highlighting (**a**) the numbers of nearest neighbors, (**b**) average, (**c**) minimum and (**d**) maximum primary dendrite arm spacings. The red/blue circles indicate microstructural changes within a growth step of ≈1mm. Red circles show positions where dendrites were eliminated by competitive growth. The blue circle shows a dendrite that has newly formed by branching. The arrows mark three misaligned dendrites. Same sample region as previously considered in Figure 5, Figure 8 and Figure 9.

An effort was made to analyze the arrangements and spacings of dendrites using tomography based optical microscopy. Figure 12 presents color-coded Voronoi plots of the numbers of nearest neighbors (Figure 12a), as well as average (Figure 12b), minimum (Figure 12c) and maximum (Figure 12d) primary dendrite arm spacings. The data were generated from the first image slice of the newly-grown SX (z_S_ = 11.4 mm). The region considered in Figure 12 again matches the sample part previously considered in Figure 5, Figure 8 and Figure 9 (see polygonal dendrite constellation). Three black arrows indicate the three misaligned dendrites. A close look at the results from Voronoi tessellation reveals that the misaligned dendrites do not significantly differ from other dendrites in terms of their numbers of nearest neighbors or local spacings. Most dendrites are characterized by six nearest neighbors, an average spacing close to 200 µm and lowest/highest spacings of about 150 µm/300 µm. The tomographic data allow to detect whether dendrites, which were present in the first image slice, were eliminated by competitive growth [59,74], or whether new dendrites have formed during solidification. The red/blue circles in Figure 12c,d indicate positions where dendrites were extinct/newly-formed within a growth step of ≈1 mm from the first to the second image slice. The data reveal that, in fact, several dendrites were eliminated by competitive growth as they are only present in the first image slice, Figure 12c. An important finding was that one of the three misaligned dendrites was not able to persist during on-going solidification, Figure 12c. While a relatively large number of dendrites was extinct during the first millimeter of solidification (red dots in Figure 12c), the formation of new dendrites (corresponding to the blue dot in Figure 12d) represents a rare phenomenon. In fact, only one single dendrite has newly formed in the region presented in Figure 12d during the first millimeter of solidification. Finally, Figure 12c,d clearly show that the formation/extinction of dendrites occurs in cross-sectional regions with lowest minimum (Figure 12c)/highest maximum (Figure 12d) dendrite spacings.

**Figure 13:**
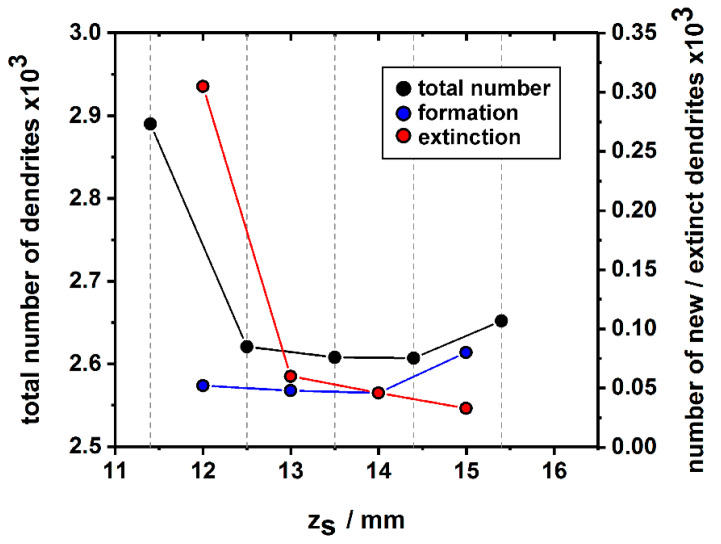
Evolution of dendrite numbers. The total number of dendrites is presented by black symbols (data values on left vertical axis). The plot shows the numbers of newly-formed (blue symbols, data values on right vertical axis) and extinct dendrites (red symbols; data values on right vertical axis) in the first 4 mm of the solidifying rod (evaluated from full cross section of the rod).

Figure 13 shows how the total number of dendrites as well as the numbers of newly-formed and extinct dendrites evolve in the *complete* cross section area of the SX bar during the first 4 mm of solidification. The corresponding data were manually retrieved from the tomographic image data sets. The numbers of newly-formed and extinct dendrites were determined by a careful evaluation of microstructural changes between adjacent image slices. Since it was not possible to precisely detect the z_S_ positions where specific formation/extinction events occured (the increment between two adjacent slices is close to 1 mm), the corresponding data were assigned to the central z_S_ position between two image slices. The cross section from the first z_S_ position, which corresponds to an early solidification stage of the newly grown crystal (Figure 2), contains a total number of 2892 dendrites. This number significantly decreased within the first incremental z_S_ step due to dendrite extinction. In fact, close to 300 dendrites did not survive competitive dendrite growth in this early stage of the solidification experiment. During subsequent solidification, the total number of dendrites remained almost constant at a value close to 2600. The further evolution is charatcerized by almost constant rates for dendrite formation and extinction that both are close to 50 dendrites per millimeter solidification growth.

**Figure 14:**
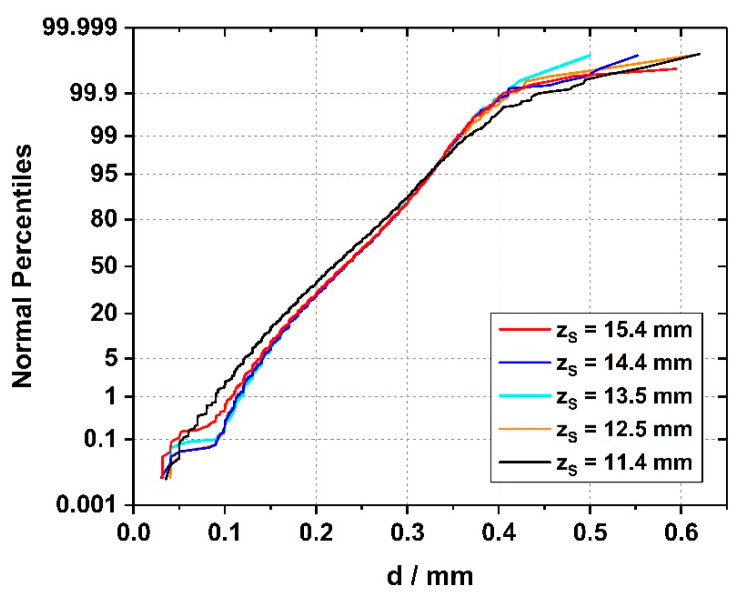
Cumulative frequency curves (probability plots) of dendrite spacings retrieved from five complete tomographic cross sections of the SX bar. Over 7000 individual spacings (one dendrite has several neighbors) were evaluated for each z_S_ position/tomographic slice.

Detailed cummulative histograms, which are based on the spacings of *all* dendrites which were detected in each 113 mm^2^ sample cross section, are presented in Figure 14. Since each dendrite has several neighbors, a total number of 8500 individual spacings was considered for the first sample slice (z_S_ = 11.4 mm) and 7694 for the second slice (z_S_ = 12.5 mm). For the second slice, 95.4% of all spacings are distributed between 117 and 346 µm, and the average dendrite spacing is close to 230 µm. The histograms presented in Figure 14 allow to conclude that the distributions of dendrite spacings for all five tomographic slices are a very similar. The only exception is the histogram from the first z_S_ = 11.4 mm position, where the majority of all spacings is slightly smaller than the data from other image slices. The observation is in line with the trends previoulsy presented in Figure 12 and Figure 13, where a reduction of the total number of dendrites was documented for the early stages of the crystal growth experiment.

### 3.4. Competitive Growth between Misoriented and Surrounding Dendrites

**Figure 15:**
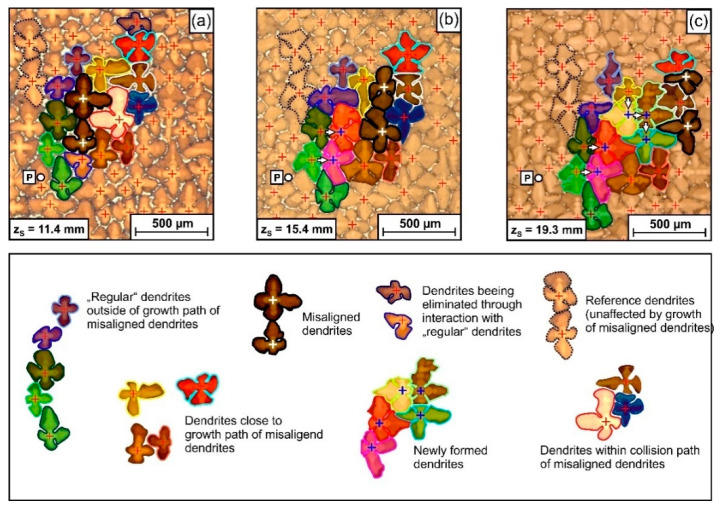
Growth interactions between two slightly misaligned dendrites (dark silhouette, white crosses) with surrounding dendrites during 8 mm of directional solidification growth. Microstructure snapshots at tomographic z_S_ positions of (**a**) 11.4 mm, (**b**) 15.4 mm and (**c**) 19.3 mm. For details, see text.

**Figure 16:**
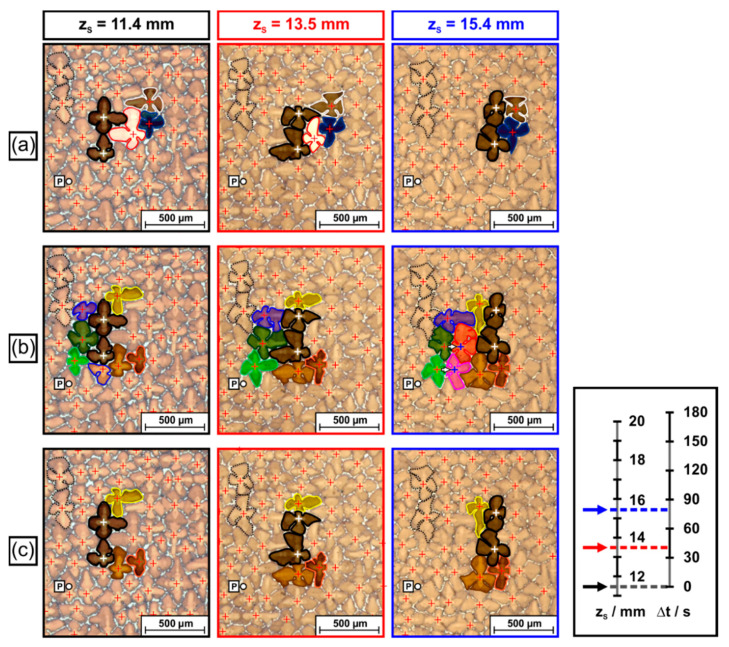
Specific aspects of interactions between dendrites, isolated from the image series in Figure 15. (**a**) Overgrowth of matrix dendrites by slightly misaligned dendrites. (**b**) Branching events and formation of tertiary dendrites behind the growth path of misaligned dendrites. (**c**) Early stages of branching of dendrites located close to the growth paths of misaligned dendrites. For details, see text.

The tomographic image data (raw data available: link in reference [66]) allow to study interactions and competitive growth between slightly misaligned dendrites and neighboring dendrites. Figure 15 presents an overview that shows how the two more severely misaligned dendrites, indicated in Figure 5, Figure 8, Figure 9, Figure 11 and Figure 12, interact with their microstructural environment. The different Figure 15a–c represent snapshots from various stages of dendrite growth at z_S_ positions of 11.4 mm (Figure 15a), 15.4 mm (Figure 15b) and 19.3 mm (Figure 15c). The image sequence in Figure 15 covers 8 mm of dendrite growth, corresponding to a time interval of 160s in the solidification experiment. All micrographs, Figure 15a–c, show exactly the same region with respect to the x_S_-y_S_ coordinates (see reference point “P”, which has identical coordinates in all images). The two misaligned dendrites are marked by black silhouettes, white crosses and dark semi-transparent shading. Different colors were used to mark dendrites with regular orientation located within, behind and sideways of the projected growth paths of the two misaligned dendrites. Two dendrites with regular orientation are indicated with dashed lines in the upper left of Figure 15a. These dendrites have the same orientation as most other dendrites in this sample region. They serve as a reference to reflect the average growth behavior. During solidification, the positions of these reference dendrites have slightly shifted towards the lower right, as the SX sample is characterized by a slight overall misorientation of 2.5°, Figure 9e and Figure 10e. As a striking observation, the slightly misaligned dendrites do not follow the overall growth trend. They both have parallel projected growth directions towards the upper right, in line with the tomographic data presented in Figure 8. Figure 15 provides direct evidence that these two dendrites overgrow other dendrites that are located within their growth paths. In addition, one can also detect direct interactions with neighboring dendrites located besides or even behind the growth paths. Since it is tedious to identify and to follow all different microstructural evolution processes in Figure 15, a different representation where only selected dendrites are marked is presented in Figure 16. The three image columns in Figure 16 are related to different tomographic z_S_ positions and thus to different time steps of the solidification experiment (first column: 11.4 mm/0 s, second column: 13.5 mm/42 s, third column: 15.4 mm/80 s) as indicated by the legend on the right of Figure 16.

Three elementary interaction processes between dendrites were identified. *First*, Figure 16a shows that the two misaligned dendrites managed to overgrow one dendrite (bright color, red silhouette) located within their growth path. This obstacle dendrite initially had relatively long secondary arms at a z_S_ height of 11.4 mm. However, the lengths of these arms decreased as the two misaligned dendrites approached (z_S_ = 13.5 mm), Figure 16a. Finally, the misaligned dendrites managed to overgrow/to extinguish the bright dendrite with regular orientation at z_S_ = 15.4 mm. *Second*, the movement of the misaligned dendrites triggered branching events from matrix dendrites located rear their growth paths. In Figure 16b, two green dendrites can be spotted. Once the two slightly misaligned dendrites move towards the upper right of the image, i.e., away from the two green dendrites, the available space increases (see situation in Figure 16b for z_S_ = 13.5mm). This process results in the growth of the secondary arms of the two green dendrites. At a later stage, new tertiary dendrites evolve from the secondary arms, as documented for a z_S_ position of 15.4 mm. The small arrows in Figure 15 and Figure 16 indicate branching directions. A *third* type of dendrite interaction process is presented in Figure 16c. The propagation of the slightly misaligned dendrites affects the secondary arm lengths of a yellow and a brown dendrite located aside of the growth paths of the two dark-shaded dendrites. In the case of the yellow dendrite, the secondary arm length first decreases (shown for a z_S_ height of 13.5 mm) and afterwards again increases (z_S_ = 15.4 mm). Finally, a new tertiary dendrite formed by branching, which is visible in the overview image, Figure 15c, at z_S_ = 19.3 mm (surrounded with bright green line). We note that the Supplementary Materials of this study contains three movies that are based on the image sequences presented in Figure 15 and Figure 16. These movies allow to follow all aspects shown in Figure 16 in detail. They also present further microstructural evolution steps from later stages of the solidification experiment.

## 4. Discussion

*Methodical aspects.* To our knowledge, this study represents the first case where the growth of a large number of individual dendrites (close to 2500) was reconstructed in detail by a post-mortem tomographic approach. Our characterization technique can be successfully applied to investigate the evolution of dendritic microstructures in single crystals or directionally solidified materials. Moreover, it allows to detect and to trace the formation of small angle misorientation defects; to evaluate aspects related to dendrite arrangements, Figure 12, and competitive dendrite growth, Figure 15; and to study direct interactions between dendrites with different orientations, Figure 16. It was demonstrated that the use of color-coded orientation distributions maps, Figure 9, Figure 10 and Figure 11, where dendrite growth directions are expressed through polar and azimuth angles, provides new microstructural information. This type of visualization allows to detect small-scale (e.g., size close to 1–3 single dendrites) or larger-scale (e.g., with sizes of several 100 dendrites) microstructural features, Figure 10.

A drawback of the applied serial sectioning approach is that a manual evaluation of dendrite positions represents a tedious and time-consuming effort. Therefore, there is a need to facilitate this process. It is reasonable to assume that the present approach will significantly benefit from the application of machine learning techniques, which are currently of interest in materials science, e.g., [75,76,77]. For example, neuronal networks could be applied to detect dendrites from metallographic cross sections, to retrieve their positions and to trace their growth across tomographic images. The feasibility of applying machine learning procedures to the analysis of dendritic microstructures was reported in [77,78]. We have recently initiated research activities in this field. One goal is to develop a reliable automated dendrite detection routine, which will allow to analyze even larger sample volumes and to retrieve additional information.

*Dendrite growth directions, crystal orientations and crystal mosaicity.*Figure 9f and Figure 11c demonstrate that morphological dendrite growth directions and crystallographic <001> directions, which represent the natural solidification growth direction for fcc metals [44], almost perfectly coincide for the solidification conditions imposed in the present study. This finding confirms that crystal mosaicity in SX is directly related to the spread of crystal orientations/growth directions, which results from possible dendrite deformation processes, [29,30,41,42,46,47,48,49,50,51]. In our previous study [30], we have documented the occurrence of continuous and sudden dendrite bending events for SX prepared with almost identical solidification conditions as is the present work. These bending events resulted in a continuous increase of the degree of mosaicity during on-going solidification. However, it is unlikely that these deformation processes are solely responsible for the mosaic microstructure observed in the present study, e.g., Figure 10, where the very early stage of a seeded crystal growth process was investigated. As sudden dendrite deformation processes appear to be rare events [30], and as continuous dendrite deformation occurs along larger distances, e.g., 25 mm [30], these processes cannot represent the main origin of the mosaicity observed in the first 4 mm solidification length considered in the present study, Figure 2a. Therefore, it is reasonable to assume that the mosaic character of the cylindrical specimen was directly inherited form the SX seed used for Bridgman solidification, Figure 1 and Figure 2. We assume especially that the long-range orientation gradients, Figure 10a, represent a feature that was directly passed-on from the SX seed material. To verify this, there is a need to apply our tomographic procedure to study mosaicity of the seed material. Furthermore, it is interesting to investigate how orientation defects evolve during the subsequent stages of our solidification experiment. These aspects will be addressed in a following study.

*Evolution of dendrite numbers and dendrite spacings.* Figure 12 and Figure 13 document that a significant decrease/increase of dendrite numbers/spacings occurred within the first solidification step of seeded Bridgman processing, Figure 1 and Figure 2. Afterward, almost constant dendrite numbers/spacings are observed, Figure 12 and Figure 13. It is known that the initiation of the withdrawal process during Bridgman crystal growth temporarily triggers the formation of a larger number of dendrites [62,79]. However, once constant solidification conditions are established, microstructures with constant dendrite spacings are maintained [80,81,82,83]. This finding is confirmed in Figure 13, where nearly the same dendrite numbers were preserved through a balance between dendrite extinction and formation. It is interesting to investigate how this balance evolves in the later stages of the solidification experiment, where the degree of crystal mosaicity is higher [30], such that more intensive growth competition between misoriented dendrites occurs.

*Misorientations and interactions between dendrites.* Competitive growth between dendrites has been of general interest in solidification research for decades, e.g., [59,74,84,85,86,87]. It represents an elementary process that governs the evolution of as-cast microstructures. Growth competition depends on misorientation angles and geometric conditions, i.e., whether converging or diverging dendrite growth occurs. In the case of converging growth, the misoriented dendrites can overgrow those with favorable orientation [59], which is less likely for diverging growth. Growth competition also depends on solidification conditions. With increasing temperature gradient and cooling rate, small tertiary dendrite branches produced from side branches of unfavorably oriented dendrites have a higher chance to evolve into new primary dendrite arms [87]. The scenario presented in Figure 15 and Figure 16 differs from what has been considered in literature, e.g., [59,74,84,85,86,87]. Most literature studies focus on the competitive growth between a large number of dendrites, which make up two different grains, one with a favorable and the other with an unfavorable orientation. The present study, however, considers a scenario where only two slightly misoriented dendrites slowly cross a field of dendrites with regular orientation, Figure 8, Figure 15 and Figure 16. It was observed (Figure 16a) that the two misaligned dendrites managed to overgrow one regularly oriented dendrite (bright dendrite in Figure 16a). This situation corresponds to what is considered as converging growth in literature, e.g., [59,74]. It is clear that, in general, the persistence of misaligned dendrites depends on local arrangements of dendrites situated within their growth paths. Figure 15 and Figure 16b,c document that isolated dendrites with small misorientations also interact with other surrounding regular dendrites. They trigger branching events from regular dendrites located to the rear of, Figure 16b, or aside of, Figure 16c, their projected growth trajectory. The formation of new dendrites by branching aims at establishing a microstructure with dendrite spacings that fall into a specific band [78,80]. The two types of branching events presented in Figure 16b,c are similar to what has been referred to as uniplanar and non-uniplanar branching by Meng et al. [59]. The present work demonstrates that the presence of dendrites with slight misorientations triggers these two types of branching events during solidification growth.

*Raw data obtained in the present study*. We provide image data (registered high-resolution micrographs of complete ingot cross sections containing several thousand dendrites) and x_S_-y_S_ position data sets of individual dendrites to the public. The data are accessible through the data repository zenodo (link in reference [66]). We hope that these data may be useful for other research activities.

## 5. Summary and Conclusions

The present study explores crystal mosaicity associated with the evolution of dendritic solidification structures during seeded Bridgman processing of single crystal Ni-base superalloys. A novel tomographic approach was applied which allowed to extract rich data sets from optical wide field micrographs sequentially taken at different height positions. The features of ≈2500 dendrites were documented in a large sample volume of 450 mm^3^. Special emphasis was placed on the early stages of crystal growth. The key findings of the present work can be summarized as follows:

(1) The novel tomographic procedure described in the present work allows to evaluate the growth behavior of a large number of dendrites accounting for different individual growth directions. The 3D results can be visualized using color-coded orientation distribution maps where dendrite growth directions are represented by polar and azimuth angles.
(2) The specimen cross sections consist of regions that frequently feature interdendrite misorientation angles less than 2°. However, a small number of isolated dendrites (0.24%) showed larger deviations in growth directions.
(3) Individual dendrites grow in crystallographic <001> directions, which was shown using the recently developed rotation vector base-line electron back scatter diffraction method (RVB-EBSD method). This confirms that crystal mosaicity is directly related to dendrite misorientations.
(4) Statistical distributions of primary dendrite arm spacings were evaluated. It was found that in the early stages of seeded Bridgman solidification, many dendrites form. Their number subsequently decreases, however, due to competitive dendrite growth as constant solidification conditions are established. The later solidification stages are characterized by a balance between the formation of new and the extinction of pre-existing dendrites.
(5) A local scenario was documented, where two slightly misoriented dendrites strongly affect the growth kinetic of their environment. These two dendrites followed their deviating growth direction and overgrew other dendrites in their growth path. The space behind these two dendrites was subsequently filled with new tertiary dendrites, branching out from secondary dendrite arms of regularly oriented surrounding dendrites from different sides. The technique presented in this work not only provides sound statistic information on the average growth behavior but also allows to study such unusual local events.

## Data Availability

All registered image data and dendrite positions for the slices from z_S_ = 11.4 mm to z_S_ = 15.4 mm are available to the public in the open-access data repository zenodo (link available in Ref. [66]).

## Supplementary Materials

The following are available online at http://www.mdpi.com/article/10.3390/ma14174904/s1, Video S1: extinction of dendrites; Videos S2 and S3: formation of new dendrites.

## References

[1] Versnyder F.L., Shank M.E. (1970). The development of columnar grain and single crystal high temperature materials through directional solidification. Mater. Sci. Eng..

[2] Meetham G.W. (1981). The Development of Gas Turbine Materials.

[3] McLean M. (1983). Directionally Solidified Materials for High Temperature Service.

[4] Durand-Charree M. (1997). The Microstructure of Superalloys.

[5] Bürgel R., Maier H.J., Niendorf T., Bürgel R., Maier H.J., Niendorf T. (2011). Hochtemperaturlegierungen. Handbuch Hochtemperatur-Werkstofftechnik.

[6] Reed R.C. (2006). The Superalloys—Fundamentals and Applications.

[7] Evans R.W., Wilshire B. (1985). Creep of Metals and Alloys.

[8] Cadek J. (1988). Creep in Metallic Materials.

[9] Pollock T.M., Tin S. (2006). Nickel-based superalloys for advanced turbine engines: Chemistry, microstructure and properties. J. Propuls. Power.

[10] Quested P.N., McLean M. (1984). Solidification morphologies in directionally solidified superalloys. Mater. Sci. Eng..

[11] Parsa A.B., Wollgramm P., Buck H., Somsen C., Kostka A., Povstugar I., Choi P.P., Raabe D., Dlouhy A., Müller J. (2015). Advanced scale bridging microstructure analysis of single crystal Ni-base superalloys. Adv. Eng. Mater..

[12] Nörtershäuser P., Frenzel J., Ludwig A., Neuking K., Eggeler G. (2015). The effect of cast microstructure and crystallography on rafting, dislocation plasticity and creep anisotropy of single crystal Ni-base superalloys. Mater. Sci. Eng. A.

[13] Pollock T.M., Murphy W.H. (1996). The breakdown of single-crystal solidification in high refractory nickel-base alloys. Metall. Mater. Trans. A.

[14] Hobbs R.A., Tin S., Rae C.M.F. (2005). A castability model based on elemental solid-liquid partitioning in advanced nickel-base single-crystal superalloys. Metall. Mater. Trans. A.

[15] Zhou Y.Z., Volek A., Singer R.F. (2005). Influence of solidification conditions on the castability of nickel-base superalloy IN792. Metall. Mater. Trans. A.

[16] Rappaz M., Rettenmayr M. (1998). Simulation of solidification. Curr. Opin. Solid State Mater. Sci..

[17] Gilman J.J. (1963). The Art and Science of Growing Crystals.

[18] Hong J.P., Ma D.X., Wang J., Wang F., Dong A.P., Sun B.D., Bührig-Polaczek A. (2015). Geometrical effect of freckle formation on directionally solidified superalloy CM247 LC components. J. Alloys Compd..

[19] Ma D.X., Zhou B., Buhrig-Polaczek A., Heilmaier M. (2011). Investigation of freckle formation under various solidification conditions. Euro Superalloys 2010.

[20] Auburtin P., Wang T., Cockcroft S.L., Mitchell A. (2000). Freckle formation and freckle criterion in superalloy castings. Metall. Mater. Trans. B.

[21] Aveson J.W., Tennant P.A., Foss B.J., Shollock B.A., Stone H.J., D’Souza N. (2013). On the origin of sliver defects in single crystal investment castings. Acta Mater..

[22] Carney C.A., Beech J., Beech J. (1997). The origin of sliver defects in single crystal turbine blades. Decennial International Conference on Solidification Processing.

[23] Yang C., Liu L., Zhao X., Zhang J., Sun D., Fu H. (2013). Formation of stray grains during directional solidification of a superalloy AM3. Appl. Phys. A.

[24] Zhou Y.Z. (2011). Formation of stray grains during directional solidification of a nickel-based superalloy. Scr. Mater..

[25] Darwin C.G. (1922). XCII. The reflexion of X-rays from imperfect crystals. Lond. Edinb. Dublin Philos. Mag. J. Sci..

[26] Bellet D. (1990). Etude Des Textures des Superalliages Monocristallins Par Diffraction et Diffusion Des Rayonnements: X, γ et Neutrons. Ph.D. Thesis.

[27] Brückner U., Epishin A., Link T. (1997). Local X-ray diffraction analysis of the structure of dendrites in single-crystal nickel-base superalloys. Acta Mater..

[28] Klam H., Blank E. The growth of modern superalloy single crystals. Proceedings of the First Conference on Advanced Materials and Processes (EUROMAT’89).

[29] Doherty R.D. (2003). Comments on “Mechanical deformation of dendrites by fluid flow during the solidification of undercooled melts”. Scr. Mater..

[30] Hallensleben P., Scholz F., Thome P., Schaar H., Steinbach I., Eggeler G., Frenzel J. (2019). On crystal mosaicity in single crystal Ni-based superalloys. Crystals.

[31] Sass V., Glatzel U., Feller-Kniepmeier M. (1996). Anisotropic creep properties of the Nickel-base superalloy CMSX-4. Acta Mater..

[32] Pollock T.M., Argon A.S. (1991). Creep resistance of CMSX-3 Nickel base superalloy single crystals. Acta Metall. Mater..

[33] Rae C.M.F., Reed R.C. (2007). Primary creep in single crystal superalloys. Acta Mater..

[34] Ram F., Li Z., Zaefferer S., Haghighat S.M.H., Zhu Z., Raabe D., Reed R.C. (2016). On the origin of creep dislocations in a Ni-base, single-crystal superalloy: An ECCI, EBSD, and dislocation dynamics-based study. Acta Mater..

[35] Harris K., Erickson G.L., Brentnall W.D., Aurrecoechea J.M., Sikkenga S.L., Kubarych K.G., Antolovich S.D. Development of the rhenium containing superalloys CMSX-4 & CM 186 LC for single crystal blade and directionally solidified vane applications in advanced turbine engines. Superalloys 1992.

[36] He J., Scholz F., Horst O.M., Thome P., Frenzel J., Eggeler G., Gault B. (2020). On the rhenium segregation at the low angle grain boundary in a single crystal Ni-base superalloy. Scr. Mat..

[37] D’Souza N., Newell M., Devendra K., Jennings P.A., Ardakani M.G., Shollock B.A. (2005). Formation of low angle boundaries in Ni-based superalloys. Mater. Sci. Eng. A.

[38] Newell M., D’Souza N., Green N.R. (2009). Formation of low angle boundaries in Ni-based superalloys. Int. J. Cast Met. Res..

[39] Newell M., Devendra K., Jennings P.A., D’Souza N. (2005). Role of dendrite branching and growth kinetics in the formation of low angle boundaries in Ni–base superalloys. Mater. Sci. Eng. A.

[40] Schaefer R., Black D., Vaudin M., Mueller B., Giamei A. Geometry and mechanisms of dendrite misalignments in superalloy single crystals. Proceedings of the 4th Decennial International Conference on Solidification Processing.

[41] Aveson J.W., Reinhart G., Nguyen-Thi H., Mangelinck-Noël N., Tandjaoui A., Billia B., Goodwin K., Lafford T.A., Baruchel J., Stone H.J. (2012). Dendrite bending during directional solidification. Superalloys 2012.

[42] Aveson J.W., Reinhart G., Nguyen-Thi H., Mangelinck-Noel N., D’Souza N., Stone H.J., Guedou J.Y., Chone J. (2014). Origins of misorientation defects in single crystal castings: A time resolved in situ synchrotron X-ray radiography study. Eurosuperalloys 2014—2nd European Symposium on Superalloys and Their Applications.

[43] Reinhart G., Nguyen-Thi H., Mangelinck-Noël N., Baruchel J., Billia B. (2014). In Situ Investigation of Dendrite Deformation During Upward Solidification of Al-7wt.%Si. JOM-US.

[44] Lee D.N., Kim K., Lee Y., Choi C.-H. (1997). Factors determining crystal orientation of dendritic growth during solidification. Mater. Chem. Phys..

[45] Mullis A.M. (1999). Growth induced dendritic bending and rosette formation during solidification in a shearing flow. Acta Mater..

[46] Uehara T., Tsujino T. (2005). Phase field simulation of stress evolution during solidification. J. Cryst. Growth.

[47] Uehara T., Fukui M., Ohno N. (2008). Phase field simulations of stress distributions in solidification structures. J. Cryst. Growth.

[48] Wagner A., Shollock B.A., McLean M. (2004). Grain structure development in directional solidification of nickel-base superalloys. Mater. Sci. Eng. A.

[49] Dragnevski K., Mullis A.M., Walker D.J., Cochrane R.F. (2002). Mechanical deformation of dendrites by fluid flow during the solidification of undercooled melts. Acta Mater..

[50] Mullis A.M., Walker D.J., Battersby S.E., Cochrane R.F. (2001). Deformation of dendrites by fluid flow during rapid solidification. Mater. Sci. Eng. A.

[51] Pilling J., Hellawell A. (1996). Mechanical deformation of dendrites by fluid flow. Metall. Mater. Trans. A.

[52] Thome P., Medghalchi S., Frenzel J., Schreuer J., Eggeler G. (2019). Ni-base superalloy single crystal (SX) mosaicity characterized by the Rotation Vector Base Line Electron Back Scatter Diffraction (RVB-EBSD) method. Ultramicroscopy.

[53] Siredey N., Boufoussi M.B., Denis S., Lacaze J. (1993). Dendritic growth and crystalline quality of nickel-base single grains. J. Cryst. Growth.

[54] Weiland H., Rouns T.N., Liu J. (1994). The role of particle stimulated nucleation during recrystallization of an aluminium-manganese alloy. Z. Metallkd..

[55] Li M., Ghosh S., Rouns T.N., Weiland H., Richmond O., Hunt W. (1998). Serial sectioning method in the construction of 3-D microstructures for particle-reinforced MMCs. Mat. Char..

[56] Sidhu R.S., Chawla N. (2004). Three-dimensional microstructure characterization of Ag_3_Sn intermetallics in Sn-rich solder by serial sectioning. Mat. Char..

[57] Spowart J.E. (2006). Automated serial sectioning for 3-d analysis of microstructures. Scr. Mater..

[58] Yokomizo T., Enomoto M., Umezawa O., Spanos G., Rosenberg R.O. (2003). Three-dimensional distribution, morphology, and nucleation site of intragranular ferrite in association with inclusions. Mater. Sci. Eng. A.

[59] Meng X.B., Lu Q., Zhang X.L., Li J.G., Chen Z.Q., Wang Y.H., Zhou Y.Z., Jin T., Sun X.F., Hu Z.Q. (2012). Mechanism of competitive growth during directional solidification of a nickel-base superalloy in a three-dimensional reference frame. Acta Mat..

[60] Liu Z., Lin M., Yu D., Zhou X., Gu Y., Fu H. (2013). Dependence of competitive grain growth on secondary dendrite orientation during directional solidification of a Ni-based superalloy. Metall. Mater. Trans. A.

[61] Hu S., Liu L., Yang W., Zhang J., Huang T., Wang Y., Zhou X. (2018). Competitive converging dendrites growth depended on dendrite spacing distribution of Ni-based bi-crystal superalloys. J. Alloys Compd..

[62] Hallensleben P., Schaar H., Thome P., Jöns N., Jafarizadeh J., Steinbach I., Eggeler G., Frenzel J. (2017). On the evolution of cast microstructures during processing of single crystal Ni-base superalloys using a Bridgman seed technique. Mat. Des..

[63] Imagic-IMS. https://imagic.ch/en/imagic-ims.

[64] CorelDRAW Graphics Suite. https://www.coreldraw.com/en/.

[65] ImageJ. https://imagej.de.softonic.com/.

[66] Scholz F., Cevik M., Hallensleben P., Thome P., Eggeler G., Frenzel J. (2021). Image and dendrite position raw-data generated in the present work. Zenodo.

[67] Tschopp M.A., Miller J.D., Oppedal A.L., Solanki K.N. (2013). Characterizing the local primary dendrite arm spacing in directionally solidified dendritic microstructures. Metall. Mater. Trans. A.

[68] Aurenahmmer F., Klein R., Sack J.-R., Urrutia J. (2000). Voronoi Diagrams. Handbook of Computational Geometry.

[69] Hielscher R., Schaeben H. (2008). A novel pole figure inversion method: Specification of the MTEX algorithm. J. Appl. Cryst..

[70] Bachmann F., Hielscher R., Schaeben H. (2010). Texture analysis with MTEX—Free and open source software toolbox. Solid State Phenom..

[71] MATLAB. https://de.mathworks.com/products/matlab.html.

[72] Takaki T., Sakane S., Ohno M., Shibuta Y., Shimokawabe T., Aoki T. (2016). Primary arm array during directional solidification of single-crystal binary alloy: Large scale phase-field study. Acta Mater..

[73] Randle V., Engler O. (2000). Introduction to Texture Analysis: Macrotexture, Microtexture and Orientation Mapping.

[74] Zhou Y.Z., Volek A., Green N.R. (2008). Mechanism of competitive grain growth in directional solidification of a nickel-base superalloy. Acta Mater..

[75] Chowdhury A., Kautz E., Yener B., Lewis D. (2016). Image driven machine learning methods for microstructure recognition. Comput. Mater. Sci..

[76] Nenchev B., Strickland J., Tassenberg K., Perry S., Gill S., Dong H. (2020). Automatic recognition of dendritic solidification structures DenMap. J. Imaging.

[77] Stan T., Thompson Z.T., Voorhees P.W. (2020). Optimizing convolution neural networks to perform semantic segmentation on large materials imaging datasets: X-ray tomography and serial sectioning. Mater. Charact..

[78] Scholz F. (2021). Metallkundliche Untersuchung zur Mosaizität in einkristallinen Nickel-Basis-Superlegierungen. Ph.D. Thesis.

[79] Wang Z., Li J., Wang J., Zhou Y. (2012). Phase field modeling the selection mechanism of primary dendritic spacing in directional solidification. Acta Mater..

[80] Steinbach I. (2008). Effect of interface anisotropy on spacing selection in constrained dendrite growth. Acta Mater..

[81] Kurz W., Fisher D.J. (1992). Fundamentals of Solidification.

[82] Dantzig J.A., Rappaz M. (2009). Solidification..

[83] Wang F., Wu Z., Ma D., Bührig-Polaczek A. (2017). Effect of directional solidification variables on the microstructures of single-crystal turbine blades of Nickel-based superalloy. Adv. Eng. Mater..

[84] D’Souza N., Ardakani M.G., Wagner A., Shollock B.A., McLean M. (2002). Morphological aspects of competitive grain growth during directional solidification of a nickel-base superalloy, CMSX4. J. Mater. Sci..

[85] Li J., Wang Z., Wang Y., Wang J. (2012). Phase-field study of competitive dendritic growth of converging grains during directional solidification. Acta Mater..

[86] Takaki T., Shimokawabe T., Ohno M., Yamanaka A., Aoki T. (2013). Unexpected selection of growing dendrites by very-large-scale phase-field simulation. J. Cryst. Growth.

[87] Hu M., Sun C., Fang H., Zhu M. (2020). Competitive dendrite growth during directional solidification of a transparent alloy: Modeling and experiment. Eur. Phys. J. E.

